# Prevalence and correlates of tobacco use among adolescents in Dhaka, Bangladesh: a cross-sectional study

**DOI:** 10.1186/s12889-026-26821-7

**Published:** 2026-02-28

**Authors:** Sahadat Hossain, Emma Beard, Dimitra Kale, Masuma Pervin Mishu, Sarah Jackson, Sharon Cox, Lion Shahab

**Affiliations:** 1 Department of Behavioural Science and Health, Institute of Epidemiology and Health Care, University College London, London, UK; 2 Department of Public Health and Informatics, Jahangirnagar University, Savar, Dhaka, Bangladesh; 3 SPECTRUM Research Consortium, Edinburgh, UK; 4 Department of Epidemiology and Public Health, Institute of Epidemiology and Health Care, University College London, London, UK; 5 Behavioural Research UK, London, Edinburgh, UK

**Keywords:** Tobacco use, Smoked tobacco, Smokeless tobacco, Second-hand smoke, Adolescents, Bangladesh

## Abstract

**Background:**

Tobacco use among adolescents in low- and middle-income countries is a public health concern. This study investigated the prevalence and correlates of ever and current tobacco use among adolescents in Dhaka, Bangladesh.

**Methods:**

A cross-sectional survey was conducted among school-going adolescents aged 11–18 years in schools across Dhaka city between June and September 2022, using an opportunistic sampling approach. A structured questionnaire collected data on socio-demographics, tobacco use behaviours, exposure to second-hand smoke (SHS), tobacco advertising, and anti-tobacco education. Logistic regression analyses were performed to identify correlates of tobacco use, adjusting for confounders.

**Results:**

A total of 2,593 adolescents participated in the survey (mean age: 15.6 years, SD = 1.6); 55.6% (*n* = 1,442) were male. Ever and current tobacco use prevalence was 16.0% (95% Confidence Interval [CI]: 14.6–17.5) and 7.3% (95% CI: 6.3–8.3), respectively. Cigarette smoking was the most common form of tobacco use (10.4%, 95% CI: 9.3–11.7, reported ever use), followed by smokeless tobacco (7.6%, 95% CI: 6.7–8.7) and non-cigarette smoked tobacco (3.3%, 95% CI: 2.7–4.0). Males had higher odds of both ever (Adjusted Odds Ratio [AOR]: 1.75, 95% CI: 1.33–2.28) and current tobacco use (AOR: 4.12, 95% CI: 2.43–6.99) compared with females. Adolescents aged 17–18 years were more likely to be current users than those aged 11–13 years (AOR: 3.27, 95% CI: 1.36–7.85). Exposure to SHS at home and tobacco advertising at points of sale were significantly associated with higher odds of both ever (AOR: 1.65, 95% CI: 1.27–2.16 and AOR: 1.61, 95% CI: 1.23–2.11, respectively) and current tobacco use (AOR: 1.75, 95% CI: 1.19–2.59 and AOR: 1.59, 95% CI: 1.07–2.36, respectively). In contrast, receiving anti-tobacco education was associated with lower odds of ever (AOR: 0.71, 95% CI: 0.52–0.99) and current tobacco use (AOR: 0.52, 95% CI: 0.33–0.84).

**Conclusion:**

Tobacco use is prevalent among school-going adolescents in urban Bangladesh. Exposure to SHS and tobacco advertising are positively associated with tobacco use, while anti-tobacco education is negatively associated, underscoring the need for strong tobacco control measures in Bangladesh.

**Supplementary Information:**

The online version contains supplementary material available at 10.1186/s12889-026-26821-7.

## Background

Tobacco use, in both smoking and smokeless forms, remains a major public health concern globally, posing significant health, economic, and social burdens. The World Health Organization (WHO) has estimated an annual death toll of over 7 million attributable to tobacco use globally, including 1.6 million non-smokers exposed to second-hand smoke (SHS) [1]. The burden of tobacco-related morbidity and mortality is particularly high in low- and middle-income countries (LMICs), where 80% of the world’s 1.3 billion tobacco users reside [1]. In South and Southeast Asia, smokeless tobacco (SLT) use presents additional challenges, contributing to over 85% of the global SLT-related burden [2]. SLT products, such as betel quid, gutkha, and khaini, are widely consumed in these regions and are associated with significant health risks, including oral cancer and other non-communicable diseases [2]. In South Asia, the use of SLT exhibits marked gendered patterns, shaped by social norms and cultural acceptability [3]. While cigarette smoking among females is often socially discouraged and underreported, SLT products are more culturally tolerated among women and adolescent girls, particularly in household and social settings [4, 5]. This gendered acceptability has been associated with a narrower gender gap in SLT use compared with smoked tobacco, underscoring the need to examine tobacco use patterns beyond smoking alone. The higher prevalence of tobacco use in LMICs is often linked to determinants such as weaker regulatory frameworks, limited public health resources, lack of interventions related to tobacco prevention and cessation, and aggressive tobacco industry marketing [6–8].

Adolescents represent a particularly vulnerable group for initiating tobacco, as they are at a stage of development marked by increased susceptibility to peer and marketing influences and higher chances of experimentation [9]. Initiating tobacco use during adolescence significantly raises the risk of longer-term, lifelong, use. The WHO estimates that approximately 38 million adolescents (25 million boys and 13 million girls) aged between 13 and 15 years currently use tobacco globally, with nearly one in every five adolescents using tobacco in some form [10]. In 2019, an estimated 155 million individuals aged 15–24 years – representing approximately 13% of this age group globally – smoked tobacco regularly, with ten countries home the largest numbers of adolescents and young smokers (China, India, Indonesia, the United States, Turkey, the Philippines, Mexico, Bangladesh, Russia, and Pakistan) collectively accounting for 55·9% of the total [11]. The prevalence of adolescent tobacco use varies widely, ranging from 2.0% to over 30.0%, depending on the region and the form of tobacco [12]. In addition, 16.4% to 85.4% of adolescents worldwide are exposed to SHS, rates are particularly high in LMICs [13]. Several environmental and contextual factors contribute to tobacco use among adolescents, including exposure to direct tobacco use by peers and parents, as well as other social influences such as SHS exposure and targeted tobacco industry marketing [14–18]. Evidence suggests that SHS exposure increases smoking susceptibility and initiation among adolescents [16, 17]. Adolescents are also directly targeted through various marketing channels, and systematic reviews consistently connect tobacco marketing with increased use of tobacco [14, 19]. Exposure to point-of-sale promotions in various settings is associated with higher tobacco use [16, 20], while support for smoke-free policies is linked to reduced tobacco use among adolescents [17, 21].

Bangladesh, a densely populated LMIC located in South Asia, is ranked among top ten countries with the highest prevalence of tobacco use [22, 23]. Bangladesh has high rates of use of both smoked and smokeless tobacco products across all age groups [3]. Adolescents in Bangladesh face particularly high risks due to the ubiquitous availability of tobacco products, weak enforcement of tobacco control measures, and high rates of SHS exposure. The Global School-based Student Health Survey conducted by WHO in 2014 reported that 9.8% of school adolescents in Bangladesh – 13.8% of boys and 2.0% of girls – currently used any tobacco product, defined as use on at least one day during the past 30 days [24]. The Global Burden of Diseases, Injuries, and Risk Factors Study (GBD) 2019 estimated that, in Bangladesh, the prevalence of tobacco smoking among individuals aged 15–24 years was 26.1% (95% uncertainty interval: 22·7–30·0) in males and 1.14% (95% uncertainty interval: 0·60–1·92) in females [11]. Furthermore, 31.1% of adolescents in both sex were exposed to tobacco smoke at home and 59.0% of adolescents were exposed to tobacco smoke inside enclosed public places [25].

Dhaka, one of the world’s most densely populated megacities, presents a unique urban context for adolescent tobacco use. Rapid urbanisation, high population density, widespread informal retail of tobacco products, and extensive exposure to point-of-sale advertising create an environment in which adolescents are disproportionately exposed to tobacco-related risks [26, 27]. Evidence suggests that enforcement of tobacco control regulations, including restrictions on sales to minors and smoke-free public spaces, is particularly challenging in large urban centres like Dhaka [26, 28]. Despite these risks, city-specific evidence on adolescent tobacco use and its correlates remains limited, with most available data reported at the national level.

Notwithstanding efforts to curb tobacco use, including the enactment of national tobacco control legislation in 2005 (updated in 2015), adolescents’ tobacco use persists as a challenge [9]. The widespread availability of tobacco products, weak enforcement of regulations, and a lack of tobacco use prevention interventions continue to contribute to this challenge [9, 23]. With an adolescent population of approximately 36 million – 22.0% of the total population [29] – Bangladesh’s future public health outlook is closely tied to curbing adolescent tobacco use. Although national surveys provide valuable insights, recent, urban-specific data on tobacco use and SHS exposure among school-going adolescents in Bangladesh remain scarce, particularly in the post-COVID-19 period. Understanding contemporary prevalence patterns and contextual determinants in Dhaka is therefore critical for informing targeted urban tobacco control strategies and adolescent-focused prevention interventions.

## Methods

### Aims

This study aimed to document the prevalence and correlates of tobacco use among adolescents in Dhaka, Bangladesh. Specific aims were:

- To describe the prevalence of tobacco use among adolescents, including smoked tobacco (e.g., cigarettes, cigars, bidis), smokeless tobacco (e.g., chewing tobacco, snuff), and concurrent use of both.
- To explore patterns of tobacco use, including the age of initiation, frequency of tobacco use, and types of tobacco products used.
- To identify socio-demographic, environmental, and social determinants (e.g., SHS exposure, tobacco advertising, and having ever received anti-tobacco education) associated with tobacco use behaviour among adolescents.

### Design

A cross-sectional study was conducted in Dhaka, Bangladesh, between June and September 2022, to assess the prevalence and correlates of tobacco use among school adolescents. This design was chosen to capture a snapshot of tobacco use behaviours and related socio-environmental determinants within a diverse set of schools, including public, private, boys-only, girls-only, and co-educational institutions, representing various socio-economic backgrounds, in a short timeframe.

### Sample and recruitment

The study included Bangladeshi school adolescents in Dhaka who met the following eligibility criteria: a student in school Years 8 to 11, aged between 11 and 18 years, and able to read and understand Bangla.

Participants for this study were recruited using an opportunistic sampling approach, where schools were chosen based on their accessibility and willingness to participate. This method was adopted due to logistical constraints and the exploratory, school-based nature of the study, which aimed to estimate the prevalence of tobacco use and examine correlates rather than to generate population-representative estimates. While opportunistic sampling may introduce potential limitations, including selection bias and reduced representativeness, several measures were undertaken to maximise sample heterogeneity and reduce systematic bias. First, to enhance the diversity of the sample and ensure the findings could be broadly applicable, a list of schools (*N* = 32) was compiled from different socio-economic areas within Dhaka city. Second, the list of schools represented the most common type of school following the National Curriculum and Textbook Board curriculum in ‘Bangla’. Third, by targeting schools that varied in terms of their catchment areas and student demographics, we aimed to capture a wide range of socio-economic backgrounds, including students from both affluent and underprivileged areas of the city. Schools were then approached and invited to participate in the study. Although these strategies cannot fully eliminate selection bias inherent to non-probability sampling, they were intended to capture a broad socio-demographic cross-section of urban school-going adolescents in Dhaka. The use of non-probability, school-based sampling limits the generalisability of prevalence estimates beyond similar urban educational settings. While this design does not permit population-representative inference, it allows examination of associations and contextual correlates within a high-risk urban environment. The findings should therefore be interpreted as context-specific and hypothesis-generating rather than definitive estimates for all adolescents in Dhaka or Bangladesh.

Of the 32 schools approached, the majority (75%, *n* = 24) agreed to participate and provided the necessary permissions to conduct the survey among their students. Subsequently, an invitation letter was sent to the parents/guardians of eligible students from the participating schools. This letter contained detailed information about the study, including the purpose, procedures, and potential risks and benefits. Along with the invitation, parents/guardians were provided with the survey link, an online parental information sheet, a participant information sheet, parental and participant consent forms, and the questionnaire itself. To ensure the ethical conduct of the study, informed consent was obtained from both the parents/guardians and the participants themselves before participation. Participants were informed about their right to withdraw from the study at any time without any consequences. Given the age of the participants, parental consent was obtained for all students in Year 8 and 9, while assent was obtained from the students themselves. An estimated 77% of eligible students participated in the survey, based on an average class size of 35, although the full student list was not accessible for verification.

### Sample size and power analysis

The sample size calculation for this study was initially based on the 2014 Global School-based Student Health Survey conducted by WHO, which reported that 9.8% (95% Confidence Interval (CI): 6.3% to 15.0%) of school adolescents in Bangladesh (aged 13 to 17) used any tobacco product at least once during the past 30 days [24]. This survey represented the most recent nationally representative, school-based estimate of adolescent tobacco use available at the time of study design. Assuming that prevalence remained stable, a sample size of approximately 1382 participants was estimated to detect the prevalence with a relative precision of 25%, which corresponds to an absolute precision of ± 2.45% points around the estimate. In practical terms, this means the 95% CI around the observed prevalence would fall between approximately 7.35% and 12.25%, providing a reasonably narrow range for inference. Based on this sample size, the study would have 90% power to detect a change in prevalence of approximately ± 3.5% points (e.g. from 9.8% to 13.3% or to 6.3%), using a one-sample proportion test [30].

### Study procedures

Data were collected online using REDCap [31, 32], hosted in the UCL Data Safe Haven, compliant with the General Data Protection Regulation. An initial draft of the survey was developed by the lead author. To achieve good validity and reliability, the questions assessing tobacco use behaviours were adapted from the Global Youth Tobacco Survey (GYTS), a standardised and widely recognised instrument developed by the WHO for assessing tobacco use prevalence among adolescents [33]. Additional items were created to assess demographic characteristics and contextual correlates relevant to the Bangladeshi context. To ensure content validity, an expert review panel comprising behavioural science specialists reviewed the drafted questionnaire in multiple rounds. The English version of the questionnaire was subsequently translated into Bangla by a bilingual expert, followed by a back-translation by two independent local university graduates. Both versions underwent further refinement to ensure conceptual equivalence and cultural appropriateness.

To further ensure the feasibility and clarity of the survey, pilot testing was conducted with a small sample of students (*n* = 8). This group was selected purposively to reflect the target population’s age range and socio-economic diversity. The pilot assessed the usability of the online platform, the comprehensibility of survey questions, and the estimated time required for completion. Feedback from participants was used to refine the survey, addressing any issues related to comprehension and cultural relevance. These refinements included simplifying some questions, clarifying ambiguous terms, and adjusting the format to improve user experience. The final English version of the questionnaire is provided as supplementary file 1.

### Measures

#### Socio-demographic information

Socio-demographic information was gathered from all respondents through both open-ended and close-ended questions, including their age, sex (female, male), religion (Islam, Hinduism, Buddhism, Christianity, no religious belief), weekly pocket money expenditure (amount in Taka), place of living (school/college hostel, private hostel, rented house with parents/friends, owned property with parents), number of siblings, and paternal and maternal education levels (no formal education, primary, secondary, higher secondary, undergraduate, graduate, doctoral).

## Tobacco use behaviours

Tobacco use was assessed using six questions directly adapted from the WHO GYTS [33].

Ever tobacco use was measured by asking: Have you ever tried or experimented with (i) cigarettes, (ii) non-cigarette smoked tobacco (e.g., pipes, cigars, waterpipes, hookah, shisha, bidis), or (iii) smokeless tobacco products (e.g., zorda with pan, tobacco leaf, gul, khaini, panmasala)? Each item had dichotomous “yes” or “no” response options.

Current tobacco use (past 30 days) was assessed by asking: How many days did you use (iv) cigarettes, (v) non-cigarette smoked tobacco, or (vi) smokeless tobacco? Each item had the following seven response options: “0 days”, “1–2 days”, “3–5 days”, “6–9 days”, “10–19 days”, “20–29 days”, and “all 30 days”.

Participants were classified based on standard WHO GYTS operational definitions as follows:

- Current tobacco user: Using ≥1 tobacco product (smoked or smokeless) within the past 30 days.
- Ever tobacco user: Having used ≥1 tobacco product in their lifetime.
- Tobacco non-user: Never used any tobacco product.

Tobacco use patterns were assessed by asking the frequency and average number of times per day tobacco was used in the past 30 days. Age of initiation was determined by asking, “How old were you when you first tried a cigarette/other tobacco product?” Additionally, reasons for tobacco initiation were assessed with the question, “What were the reasons behind your tobacco initiation?”, with response options including curiosity, perceived attractiveness, peer influence, family tobacco use, family conflict, school factors, poor academic achievement, low self-esteem, anxiety, depression, stress, tobacco availability, exposure to advertisements at points of sale, media portrayals of tobacco use, and an open-ended “other” option.

### Exposure to SHS

Exposure to SHS was defined as being exposed to tobacco smoke on at least one occasion during the past 7 days [25, 34]. The assessment of exposure to SHS was based on three questions: “During the past 7 days, on how many days have people smoked in your presence – (i) inside your home (ii) inside any enclosed public place, other than your home (such as: school, shops, restaurants, shopping malls, movie theatres, any office, inside bus, inside train) and (iii) at any outdoor public place (such as: playgrounds, sidewalks, entrance to buildings, parks, beaches, bus terminal, railway station)?”

### Exposure to tobacco advertisement and/or promotion

Tobacco advertisement and/or promotion exposure was assessed through six questions: “During the past 30 days, did you see any advertisements and/or promotions for tobacco products (i) in retailers e.g., stores, shops, street vendors), (ii) on online social media (e.g., Facebook, Instagram, YouTube, Twitter), or (iii) see any people using tobacco on TV, in videos, or movies?”

The promotion of tobacco-branded items was assessed by asking, (iv) “Would you ever use or wear something that has a tobacco company or tobacco product name or picture on it (such as a lighter, T-shirt, hat, or sunglasses)? and (v) “Do you have something with a tobacco product brand logo on it (for example, T-shirt, pen, backpack)?” Additionally, offering a complementary product as part of a tobacco promotion was assessed with the question, (vi) “Has a person working for a tobacco company ever offered you a free tobacco product?”

Unlike the tobacco use behaviour and SHS, the six items used to assess exposure to tobacco advertisement and/or promotion collectively reflect a broader construct. Therefore, psychometric testing was performed. Exploratory factor analysis indicated that factor analysis was appropriate (Kaiser-Meyer-Olkin value = 0.63; Bartlett’s Test of Sphericity, *p* < 0.01). This supports the validity of treating these items as indicators of a latent construct.

### Tobacco education and regulatory knowledge

Participants’ exposure to health education on the risks of tobacco use and their knowledge of the country’s tobacco control rules were assessed through two questions: “Did anyone tell you about the risks of tobacco use or provide anti-tobacco education?” with “Yes” or “No” responses. The second asked participants to rate their agreement with the following statement “I have knowledge about the rules and regulations for smoking and tobacco products use (control) in Bangladesh,” in a four-point Likert scale from “Strongly agree” to “Strongly disagree”. For analysis, responses were dichotomised: “Strongly agree” and “Agree” were coded as “Yes”, while “Disagree” and “Strongly disagree” were coded as “No”.

While internal consistency testing of these two items is not applicable, we additionally assessed knowledge using six true/false statements. Exploratory factor analysis demonstrated suitability (Kaiser-Meyer-Olkin value = 0.75; Bartlett’s Test of Sphericity, *p* < 0.01), supporting the appropriateness of the knowledge measure.

### Data analysis

The expectation-maximisation method [35] was used to address missing data, with convergence achieved after 10 iterations. For most variables, the percentage of missing values was less than 5%, while for two explanatory variables (tobacco education and regulatory knowledge), it was less than 10%. The assumption that the missing data were completely at random (MCAR) was supported by Little’s MCAR test [36], which was non-significant (*p* > 0.05); accordingly, multiple imputation was not performed. Although Little’s MCAR test did not indicate systematic missingness, this test cannot definitively rule out missing-at-random or missing-not-at-random mechanisms, particularly for behavioural and knowledge-related variables. Given the low overall proportion of missing data and the descriptive, cross-sectional aims of the study, expectation–maximisation was adopted as a pragmatic approach. However, we acknowledge that this decision may underestimate uncertainty compared with multiple imputation, and results should be interpreted accordingly. The analysis plan and the protocol of this manuscript were preregistered on the Open Science Framework and can be accessed at https://osf.io/pvduj/. Data were analysed using STATA version 18.5 and SPSS version 29. Descriptive statistics summarised participants’ sociodemographic characteristics and tobacco use behaviours. Reasons for tobacco initiation were analysed as individual binary items and reported descriptively, rather than being grouped thematically, to preserve the specificity of self-reported motivations. Means with standard deviation (SD) were calculated for continuous variables, such as age, while categorical variables (e.g., sex, grade) were presented as percentages and frequencies. Bivariate analyses were conducted using chi-square (χ²) tests for categorical variables and ANOVA test for continuous variables to assess associations between sociodemographic variables and tobacco use status (ever and current tobacco use). We also checked the assumption of proportional odds using the Brant test [37]. Sensitivity analyses were performed to assess the robustness of findings by exploring alternative categorisations of ordinal variables. Multicollinearity was evaluated using variance inflation factors, with all values < 1.5, and regression diagnostics, including residuals and leverage, were inspected to ensure model fit. Dichotomisation of ordinal responses was employed to simplify interpretation and maintain consistency with established GYTS methodology. When the proportional odds assumption was violated, adjacent categories were combined into binary outcomes for valid logistic regression modelling. To identify determinants independently associated with ever and current tobacco use, univariable and multivariable logistic regression analyses were conducted. Covariates included in the multivariable models were selected based on prior evidence from the literature and empirical associations identified in the univariable analyses, in order to balance theoretical relevance with model parsimony and reduce residual confounding. The models were adjusted for age, sex, living accommodation, monthly pocket money expenditure, mother’s educational level, variables related to exposure to second-hand smoke, advertisement or promotion, and tobacco education and regulatory knowledge. Both unadjusted odds ratios (ORs) and adjusted odds ratios (AORs) with 95% CI were reported. Separate logistic regression models were run for smoked and smokeless tobacco use. All statistical tests were two-sided, with a *p*-value of < 0.05 considered statistically significant.

## Results

### Participants’ characteristics

A total of 2,593 adolescents participated in the study. The mean age of the participants was 15.6 (± 1.6) years and 55.6% (*n* = 1442) were male (Table 1). Participants were predominantly from school grade 11 (50.3%, *n* = 1303), identified as Muslim (91.9%, *n* = 2383), and had two or more siblings (53.1%, *n* = 1378). The majority of participants’ fathers had a university-level education (65.3%, *n* = 1693), while most participants’ mothers had an education level up to year 12 (53.7%, *n* = 1393). Around half of participants lived in a rented house with parents (54.2%, *n* = 1406) and had low monthly pocket money expenditures (49.4%, *n* = 1280).

**Table 1:** Participants’ sociodemographic characteristics distributed by tobacco use status (*N* = 2,593)

Characteristics	Total, % (*n*)	Ever tobacco user ^α^			Current tobacco user		
		Yes, % (*n*)	No, % (*n*)	χ^2^-value (*p*-value)	Yes, % (*n*)	No, % (*n*)	χ^2^-value (*p*-value)
School grade							
8	15.4 (400)	6.0 (25)	17.2 (375)	49.05 (< 0.001)	5.3 (10)	16.2 (390)	35.35 (< 0.001)
9	13.7 (354)	10.8 (45)	14.2 (309)		9.0 (17)	14.0 (337)	
10	20.7 (536)	19.5 (81)	20.9 (455)		15.4 (29)	21.1 (507)	
11	50.3 (1303)	63.6 (264)	47.7 (1039)		70.2 (132)	48.7 (1171)	
Age (in years)							
11–13	12.5 (324)	5.3 (22)	13.9 (302)	61.66 (< 0.001)	3.2 (6)	13.2 (318)	75.04 (< 0.001)
14–16	50.0 (1296)	41.2 (171)	51.7 (1125)		30.3 (57)	51.5 (1239)	
17–18	37.5 (973)	53.5 (222)	34.5 (751)		66.5 (125)	35.3 (848)	
Mean age (± SD)	15.6 (± 1.6)	16.2 (± 1.5)	15.5 (± 1.7)	(< 0.001)	16.5 (± 1.4)	15.5 (± 1.6)	(< 0.001)
Sex							
Male	55.6 (1442)	73.3 (304)	52.2 (1138)	62.29 (< 0.001)	88.8 (167)	53.0 (1275)	90.61 (< 0.001)
Female	44.4 (1151)	26.7 (111)	47.8 (1040)		11.2 (21)	47.0 (1130)	
Religion							
Muslim	91.9 (2383)	90.4 (375)	92.2 (2008)	1.57 (0.210)	88.8 (167)	92.1 (2216)	2.57 (0.109)
Other	8.1 (210)	9.6 (40)	7.8 (170)		11.2 (21)	7.9 (189)	
Number of siblings							
0–1	46.9 (1215)	43.9 (182)	47.4 (1033)	1.79 (0.181)	43.1 (81)	47.2 (1134)	1.16 (0.282)
≥ 2	53.1 (1378)	56.1 (233)	52.6 (1145)		56.9 (107)	52.8 (1271)	
Father’s educational level							
No university	34.7 (900)	36.1 (150)	34.4 (750)	0.45 (0.503)	36.2 (68)	34.6 (832)	0.19 (0.662)
University education	65.3 (1693)	63.9 (265)	65.6 (1428)		63.8 (120)	65.4 (1573)	
Mother’s educational level							
No university	53.7 (1393)	54.4 (1185)	50.1 (208)	2.58 (0.108)	46.8 (88)	54.3 (1305)	3.90 (0.048)
University education	46.3 (1200)	45.6 (993)	49.9 (207)		53.2 (100)	45.7 (1100)	
Living accommodation							
Hostel & Other	8.6 (224)	13.5 (56)	7.7 (168)	25.01 (< 0.001)	18.1 (34)	7.9 (190)	46.96 (< 0.001)
Owned house with parents	37.1 (963)	41.9 (174)	36.2 (789)		49.5 (93)	36.2 (870)	
Rented house with parents	54.2 (1406)	44.6 (185)	56.1 (1221)		32.4 (61)	55.9 (1345)	
Monthly pocket money expenditure (taka)							
Low (< 500)	49.4 (1280)	29.4 (122)	53.2 (1158)	88.10 (< 0.001)	19.7 (37)	51.7 (1243)	95.07 (< 0.001)
Lower middle (501–1500)	21.8 (566)	28.2 (117)	20.6 (449)		27.7 (52)	21.4 (514)	
Middle (1501–3000)	16.3 (422)	20.7 (86)	15.4 (336)		22.3 (42)	15.8 (380)	
High (> 3000)	12.5 (325)	21.7 (90)	10.8 (235)		30.3 (57)	11.1 (268)	

^α^All current tobacco users were a subset of ever users; among those who had ever used tobacco, 45.3% reported current use at the time of the survey

### Prevalence of tobacco use

**Fig. 1:**
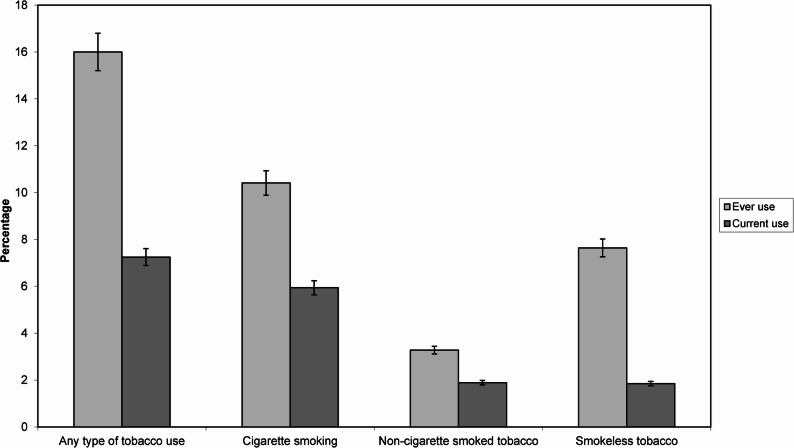
Prevalence of ever and current tobacco use of school adolescents in Dhaka, Bangladesh

The prevalence of current tobacco use among adolescents was 7.3% (95% CI: 6.3–8.3), with cigarette smoking being the most common form (5.9%, 95% CI: 5.1–6.9) (Fig. 1). The prevalence of non-cigarette smoked tobacco (e.g., cigars, waterpipes, bidis) and smokeless tobacco use were both 1.9% (95% CI: 1.4–2.5). Ever tobacco use was reported by 16% (95% CI: 14.6–17.5), with cigarette smoking having a prevalence of 10.4% (95% CI: 9.3–11.7), followed by smokeless tobacco (7.6%, 95% CI: 6.7–8.7) and non-cigarette smoked tobacco (3.3%, 95% CI: 2.7–4.0).

### Patterns of tobacco use

The mean age of tobacco initiation among school adolescents was 13.4 (± 2.9) years, with 44.7% (*n* = 84) starting between the ages of 13 and 15 (Table 2). Users of smokeless tobacco had a lower mean initiation age of 10.8 (± 2.9) years, compared to cigarette smokers, who had a mean initiation age of 13.9 (± 2.8) years. Cigarette smokers reported more frequent use on a monthly basis, but relatively less frequent daily use compared to other tobacco products. Specifically, 14.2% (*n* = 22) of cigarette smokers used tobacco more than 10 times per day, whereas this was 32.6% (*n* = 16) for users of non-cigarette smoked tobacco and 20.9% (*n* = 10) for smokeless tobacco users.

**Table 2:** Age of initiation and patterns of current tobacco use among school adolescents in Dhaka, Bangladesh, distributed by types of tobacco (*n* = 188)

Characteristics	Total, % (*n*)	Cigarette smoking, % (*n*)	Non-cigarette smoked tobacco,^a^ % (*n*)	Smokeless tobacco,^b^ % (*n*)
Initiation age				
≤ 9 years	12.8 (24)	9.1 (14)	28.6 (14)	35.4 (17)
10–12 years	17.6 (33)	13.0 (20)	18.4 (9)	37.5 (18)
13–15 years	44.7 (84)	48.7 (75)	46.9 (23)	20.8 (10)
≥ 16 years	25.0 (47)	29.2 (45)	6.1 (3)	6.3 (3)
Mean age of initiation (± SD)	13.4 (± 2.9)	13.9 (± 2.8)	11.7 (± 3.4)	10.8 (± 2.9)
Monthly tobacco use (in days)				
1–2 days	28.2 (53)	22.1 (34)	34.7 (17)	45.8 (22)
3–9 days	19.1 (36)	17.5 (27)	18.3 (9)	22.9 (11)
10–19 days	12.2 (23)	14.3 (22)	8.2 (4)	4.2 (2)
20–29 days	9.0 (17)	10.4 (16)	8.2 (4)	6.2 (3)
Everyday	31.4 (59)	35.7 (55)	30.6 (15)	20.8 (10)
Daily tobacco use (times per day)				
Non-daily	33.5 (63)	29.9 (46)	24.5 (12)	50.0 (24)
One time	19.1 (36)	16.9 (26)	26.5 (13)	20.8 (10)
2–10 times	33.0 (62)	39.0 (60)	16.3 (8)	8.4 (4)
> 10 times	14.4 (27)	14.2 (22)	32.6 (16)	20.9 (10)

^a^Non-cigarette smoked tobacco were pipes, cigars, waterpipes, hookah, shisha, bidis

^b^Smokeless tobacco products were zorda with pan, tobacco leaf, gul, khaini, panmasala

### Self-reported reasons for tobacco initiation

**Fig. 2:**
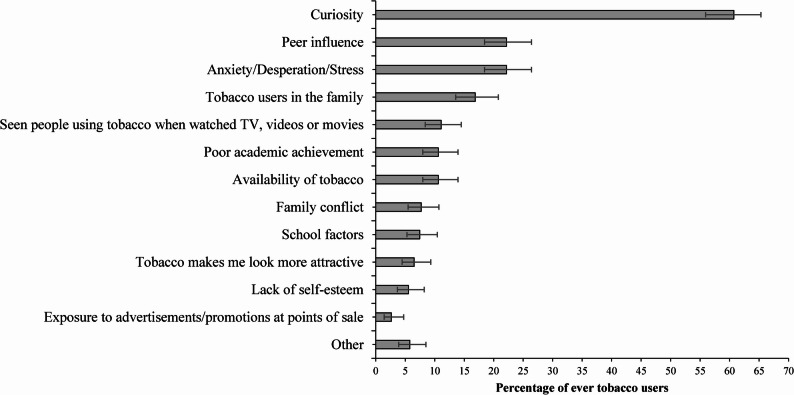
Self-reported reasons for tobacco initiation of school adolescents in Dhaka, Bangladesh

In terms of self-reported reasons for tobacco initiation (Fig. 2), curiosity was the most commonly reported factor (60.7%, 95% CI: 55.9–65.3), followed by peer influence and coping strategy to deal with anxiety/depression/stress (22.2%, 95% CI: 18.4–26.4 for each), having tobacco users in the family (16.9%, 95% CI: 13.6–20.8), media exposure (11.1%, 95% CI: 8.4–14.5), poor academic achievement (10.6%, 95% CI: 8.0–14.0), and tobacco availability (10.6%, 95% CI: 8.0–14.0). While the point estimates are slightly different, the overlap suggests no significant difference between these factors. Other self-reported reasons for tobacco initiation – such as family conflict, school-related factors, the belief that tobacco use enhances attractiveness, lack of self-esteem, and exposure to advertisements or promotions at points of sale – were each reported by fewer than 10% of participants. Stratified analyses by sex indicated significant differences in selected self-reported reasons for tobacco initiation. Peer influence was reported more frequently by males than females (25.0% vs. 14.4%, *p* = 0.022). Similarly, initiation attributed to anxiety, desperation, or stress was substantially higher among males (26.3%) compared with females (10.8%, *p* = 0.001). In contrast, initiation influenced by family tobacco use was markedly more common among females than males (*p* < 0.001). No statistically significant sex differences were observed for curiosity, media exposure, tobacco availability, or school-related factors (Supplementary Table S1).

### Correlates of tobacco use

Tobacco use was more prevalent among older students, males, those living away from family, and students with greater pocket money. For instance, current tobacco use rose from 1.9% (*n* = 6) among students aged 11–13 years to 12.8% (*n* = 125) among those aged 17–18 years. Males had a markedly higher prevalence of both ever (21.1%, *n* = 304) and current (11.6%, *n* = 167) tobacco use compared to females (9.6%, *n* = 111 and 1.8%, *n* = 21, respectively). Similarly, students living in hostels and those with higher monthly pocket money (> 3000 taka) showed substantially greater use than their peers (Table 1).

The multivariable logistic regression analysis, adjusting for all significant predictors identified in univariable logistic regression analyses, revealed significant associations between demographic characteristics, exposure to SHS, advertisement or promotion, anti-tobacco education, and tobacco use behaviour among school adolescents (Table 3). Several associations observed in unadjusted models were attenuated after adjustment, indicating confounding by overlapping sociodemographic and environmental factors. Male adolescents had 1.75 times higher odds of ever using tobacco and over four times higher odds of current tobacco use compared to females. Adolescents aged 17–18 years had 2.64 times the odds of ever using tobacco and 3.27 times the odds of current use compared to those aged 11–13 years. Living in a hostel or similar accommodation was also significantly associated with increased odds of tobacco use. Adolescents in the “middle and high” pocket money group (> 1,500 taka per month) had significantly higher odds of both ever (AOR: 1.45, 95% CI: 1.12–1.87, *p* = 0.005) and current (AOR: 1.68, 95% CI: 1.16–2.43, *p* = 0.006) tobacco use compared to those in the “low and lower middle” group.

**Table 3:** Association of sociodemographic characteristics and key correlates with any tobacco use behaviour among school adolescents in Dhaka, Bangladesh

Variable	Ever tobacco use ^a, α^				Current tobacco use ^b^			
	Unadjusted model		Adjusted model ^c^		Unadjusted model		Adjusted model ^d^	
	OR (95% CI)	*p*-value	AOR (95% CI)	*p*-value	OR (95% CI)	*p*-value	AOR (95% CI)	*p*-value
Demographics								
Sex								
Male	2.50 (1.98–3.16)	< 0.001	1.75 (1.33–2.28)	< 0.001	7.05 (4.45–11.17)	< 0.001	4.12 (2.43–6.99)	< 0.001
Female	1.00		1.00		1.00		1.00	
Age (in years)								
17–18	4.06 (2.57–6.42)	< 0.001	2.64 (1.59–4.39)	< 0.001	7.81 (3.41–17.90)	< 0.001	3.27 (1.36–7.85)	0.008
14–16	2.09 (1.32–3.31)	0.002	1.80 (1.09–2.98)	0.022	2.44 (1.04–5.11)	0.040	1.41 (0.58–3.45)	0.450
11–13	1.00		1.00		1.00		1.00	
Living accommodation								
Hostel & other	2.20 (1.57–3.09)	< 0.001	1.42 (0.96–2.11)	0.082	3.95 (2.53–6.16)	< 0.001	2.53 (1.47–4.35)	< 0.001
Owned house with parents	1.46 (1.16–1.82)	0.001	1.32 (1.02–1.71)	0.034	2.36 (1.69–3.29)	< 0.001	2.34 (1.56–3.52)	< 0.001
Rented house with parents	1.00		1.00		1.00		1.00	
Monthly pocket money expenditure								
Middle and high	2.07 (1.67–2.57)	< 0.001	1.45 (1.12–1.87)	0.005	3.02 (2.23–4.07)	< 0.001	1.68 (1.16–2.43)	0.006
Low and lower middle	1.00		1.00		1.00		1.00	
Father’s educational level								
University education	0.93 (0.75–1.16)	0.503			0.93 (0.69–1.27)	0.662		
No university	1.00				1.00			
Mother’s educational level								
University education	1.19 (0.96–1.47)	0.109			1.35 (1.00–1.82)	0.049	1.54 (1.07–2.22)	0.021
No university	1.00				1.00		1.00	
Exposure to SHS, advertisement, or promotion								
Exposed to SHS at home								
Yes	2.19 (1.73–2.77)	< 0.001	1.65 (1.27–2.16)	< 0.001	2.61 (1.89–3.60)	< 0.001	1.75 (1.19–2.59)	0.005
No	1.00		1.00		1.00		1.00	
Exposed to SHS in an enclosed public place								
Yes	1.94 (1.42–2.66)	< 0.001	1.54 (1.04–2.27)	0.030	2.21 (1.36–3.60)	0.001	1.51 (0.80–2.87)	0.207
No	1.00		1.00		1.00		1.00	
Exposed to SHS in an outdoor public place								
Yes	1.87 (1.33–2.64)	< 0.001	1.11 (0.73–1.68)	0.621	2.86 (1.58–5.20)	< 0.001	1.77 (0.82–3.80)	0.145
No	1.00		1.00		1.00		1.00	
Witnessed tobacco use in/outside of school premises								
Yes	1.93 (1.55–2.41)	< 0.001	1.21 (0.94–1.56)	0.138	2.53 (1.82–3.51)	< 0.001	1.36 (0.92–2.02)	0.122
No	1.00		1.00		1.00		1.00	
Observed tobacco use on TV, in videos, or in movies								
Yes	2.16 (1.64–2.85)	< 0.001	1.52 (1.11–2.09)	0.009	2.57 (1.66–3.96)	< 0.001	1.80 (1.06–3.05)	0.030
No	1.00		1.00		1.00		1.00	
Exposed to point-of-sale tobacco advertisement or promotion								
Yes	2.31 (1.84–2.90)	< 0.001	1.61 (1.23–2.11)	< 0.001	2.67 (1.95–3.67)	< 0.001	1.59 (1.07–2.36)	0.021
No	1.00		1.00		1.00		1.00	
Exposed to online (social media platforms) tobacco advertisement or promotion								
Yes	1.56 (1.20–2.03)	< 0.001	0.82 (0.60–1.13)	0.224	1.90 (1.33–2.70)	< 0.001	0.74 (0.47–1.18)	0.205
No	1.00		1.00		1.00		1.00	
Ever used non-tobacco products with tobacco company or tobacco product name, brand logo or picture on it								
Yes	3.44 (2.38–4.99)	< 0.001	1.59 (0.95–2.67)	0.078	4.74 (3.06–7.33)	< 0.001	1.88 (0.95–3.37)	0.070
No	1.00		1.00		1.00		1.00	
Currently using non-tobacco products with tobacco company or tobacco product name, brand logo or picture on it								
Yes	3.51 (2.28–5.39)	< 0.001	1.21 (0.66–2.22)	0.536	4.78 (2.90–7.86)	< 0.001	1.42 (0.63–3.16)	0.396
No	1.00		1.00		1.00		1.00	
Got offer and/or received complimentary tobacco products								
Yes	3.32 (2.30–4.78)	< 0.001	1.56 (1.02–2.41)	0.042	5.65 (3.72–8.57)	< 0.001	2.09 (1.24–3.55)	0.006
No	1.00		1.00		1.00		1.00	
Tobacco education and regulatory knowledge								
Have received health education on the risks of tobacco use								
Yes	0.69 (0.51–0.92)	0.012	0.71 (0.52–0.99)	0.042	0.57 (0.38–0.85)	0.006	0.52 (0.33–0.84)	0.006
No	1.00		1.00		1.00		1.00	
Have knowledge about country’s tobacco control rules and regulations								
Yes	0.48 (0.37–0.61)	< 0.001	0.57 (0.43–0.75)	< 0.001	0.48 (0.34–0.69)	< 0.001	0.67 (0.45–1.02)	0.060
No	1.00		1.00		1.00		1.00	

*OR* odds ratio, *AOR* adjusted odds ratio, *CI* confidence interval, *SHS* second hand smoke

^a^ Estimates are based on binary logistic regression with ever tobacco use (any form of tobacco) as dependent variable

^α^All current tobacco users were a subset of ever users; among those who had ever used tobacco, 45.3% reported current use at the time of the survey

^b^ Estimates are based on binary logistic regression with current tobacco use (any form of tobacco) as dependent variable

^c^ Adjusted for all significant variables in the unadjusted model of ever tobacco use

^d^ Adjusted for all significant variables in the unadjusted model of current tobacco use

SHS exposure at home significantly increased the odds of both ever (1.65 times) and current tobacco use (1.75 times). While exposure to online social media tobacco advertisements or promotions was not significantly associated with ever or current tobacco use, witnessing tobacco use in media (e.g., TV, videos, or movies) and encountering point-of-sale advertisements were both strongly associated with increased ever and current tobacco use. Receiving complimentary tobacco products was associated with 1.56 times higher odds of ever using tobacco and 2.09 times higher odds of current use. Conversely, receiving health education on the risks of tobacco use was associated with 29% lower odds of ever using tobacco and nearly 50% lower odds of current tobacco use.

In addition, the associations between study variables and smoked and smokeless tobacco use were examined separately and are presented in Supplementary Tables S2 and S3. Overall, the patterns of association were broadly consistent with those observed for any tobacco use, particularly with respect to sociodemographic characteristics, exposure to SHS at home, witnessing tobacco use in or around school premises, and exposure to point-of-sale tobacco advertising. However, some differences were observed. Male sex and older age (17–18 years) were more strongly associated with smoked tobacco use, whereas these associations were weaker or not statistically significant in relation to smokeless tobacco use. Finally, receipt of health education on the harms of tobacco was more consistently associated with lower odds of smokeless tobacco use, particularly current use.

## Discussion

This study investigated the prevalence and correlates of smoked and smokeless tobacco use among school-going adolescents in Dhaka, Bangladesh. The findings offer valuable insights into patters of tobacco use and the sociodemographic and social-environmental correlates associated with tobacco experimentation and current use in this population. Tobacco use remains widespread in Bangladesh, and adolescence represents a particularly vulnerable period for initiation [9, 38]. In the present study, 16% of adolescents reported having ever used tobacco, with higher prevalence among males. While male predominance in tobacco use is consistent with cultural norms observed across many Muslim-majority countries [39–42], the absence of a significant gender difference in smokeless tobacco use is noteworthy. This pattern likely reflects the broader cultural acceptance and accessibility of smokeless tobacco products in South Asia [43, 44], where low cost, ease of access, and limited awareness of health risks facilitate use across genders [5, 45]. Moreover, because smokeless tobacco use is less visible than smoking, it may be more easily concealed and therefore more socially acceptable for females in contexts where public tobacco use by women is culturally stigmatised.

The overall prevalence of tobacco use observed in this study was relatively higher than that reported in the most recent Global Youth Tobacco Survey in Bangladesh [25]. This comparison should be interpreted cautiously, as the present study drew on a school-based, urban sample from Dhaka and is not nationally representative. Differences in sampling strategy, geographic coverage, and study design may partially explain this discrepancy. In addition, participating schools may have differed systematically from those that declined, potentially introducing selection bias. Nevertheless, the observed prevalence highlights that tobacco use among urban adolescents remains a significant public health concern.

The notably early mean age of tobacco initiation, particularly for smokeless tobacco, warrants careful consideration. Initiation at around 10–11 years of age suggests exposure occurring well before adolescence and points to influences operating within domestic or close social environments rather than peer networks alone. This interpretation is supported by additional analyses showing that approximately three-quarters (75.7%) of smokeless tobacco users reported initiation influenced by family tobacco use. These findings are consistent with evidence from South Asia demonstrating the central role of household norms and intergenerational modelling in shaping early uptake of smokeless tobacco [46, 47]. In Bangladesh and the wider region, smokeless tobacco is comparatively normalised within household and social settings, including among women, in contrast to smoked tobacco, which is often socially proscribed for females [48–50]. Such normative environments may facilitate early exposure through routine observation and implicit endorsement by older family members, rending initiation both visible and socially acceptable within home. The strikingly early initiation age observed in this study therefore appears to reflect broader sociocultural dynamics rather than isolated individual choice.

Participants most frequently cited curiosity and peer influence as reasons for initiating tobacco use, in line with previous research highlighting the importance of social influences in adolescent tobacco uptake [23, 39, 41, 51]. Increasing age was also associated with higher prevalence of tobacco use, consistent with evidence suggesting a progression of risk across adolescence [23, 52, 53]. Given that early initiation is strongly associated with sustained use and reduced likelihood of cessation [52, 54], adolescence represents a critical window for preventive intervention. Unlike other behavioural risk factors such as diet, obesity or hypertension, tobacco use has a sensitive developmental period: individuals who do not become regular users by the age of 25 have substantially lower odds of initiating later in life [11]. Importantly, sex-stratified analyses revealed distinct gendered pathways into tobacco use. Male students were significantly more likely to report peer influence and initiation driven by anxiety, desperation, or stress, suggesting that tobacco use among boys may be more closely linked to social reinforcement and psychosocial coping mechanisms during adolescence. In contrast, initiation influenced by family tobacco use was markedly more prevalent among females. This pattern likely reflects the culturally embedded acceptability of smokeless tobacco within household settings in Bangladesh and the wider South Asian context, where such products are commonly used by adult family members and are less socially sanctioned for females than smoked tobacco [48, 50]. Routine exposure within the home, coupled with implicit social endorsement, may normalise smokeless tobacco use for girls from an early age, facilitating initiation through intergenerational modelling rather than peer-driven experimentation. Taken together, these findings highlight that adolescent tobacco initiation in this setting is not a uniform process but is shaped by gendered pathways operating across family, social, and psychological domains. Prevention strategies should therefore move beyond generic school-based messaging to incorporate family-centred and gender-sensitive approaches, particularly those addressing household tobacco norms and stress-related vulnerabilities.

Socioeconomic factors also appeared to influence tobacco use. Adolescents reporting higher levels of monthly pocket money were more likely to use tobacco, possibly reflecting greater purchasing power and access. This finding aligns with existing literature linking disposable income to adolescent risk behaviours [55]. Several environmental and contextual factors were associated with increased tobacco use, notably exposure to SHS in homes and public spaces. Similar associations have been documented in other LMICs, where SHS exposure has been shown to increase susceptibility to initiation [13, 56]. Although Bangladesh is a signatory to the WHO Framework Convention on Tobacco Control, enforcement of smoke-free policies remains uneven [38]. Strengthening smoke-free environments, particularly within households, may therefore reduce both exposure and normative acceptance of tobacco use among adolescents.

Consistent with findings from previous Global Youth Tobacco Surveys in different South Asian countries [56], our study also found that exposure to tobacco-related advertisements – both in mass media and at points of sale – was significantly associated with both ever and current tobacco use. This mirrors global evidence demonstrating the influence of point-of-sale marketing on youth tobacco uptake [19, 57]. Despite partial restrictions on tobacco advertising in Bangladesh, enforcement remains weak, especially in urban areas where promotional materials are widespread. Extending restrictions to include digital platforms frequently accessed by adolescents, alongside more robust enforcement mechanisms, could strengthen national tobacco control efforts. Experiences from countries such as Australia and the United Kingdom illustrate the effectiveness of comprehensive advertising bans in reducing youth tobacco use [58].

Finally, adolescents who reported greater awareness of tobacco control laws and exposure to anti-tobacco education were less likely to use tobacco. These findings are consistent with evidence that regulatory knowledge can act as a deterrent [59] and that well designed school-based programmes can reduce initiation [60–63]. Programmes that incorporate social competence and social influence components appear particularly effective [64]. Investing in contextually appropriate, school-based prevention initiatives, alongside broader structural measures, may therefore offer a feasible and sustainable strategy for reducing adolescent tobacco use in Bangladesh.

### Strengths and limitations

This study benefits from a substantial sample size, which enhances the reliability of the findings and allows for precise estimation of tobacco use prevalence among school-going adolescents in Dhaka. Post-hoc assessment confirmed that the achieved sample size provided sufficient precision to estimate prevalence across a range of plausible values, including both higher and lower levels than previously reported. While the sample may not be fully representative of all adolescents in the city, it includes a diverse group of participants from different school types, providing valuable insights into this population. Inclusion of both smoked and smokeless tobacco provides a more comprehensive picture of adolescent tobacco behaviours in a South Asian context. However, several limitations should be noted. While the online format facilitated secure and efficient data collection, potential limitations, such as unequal access to reliable internet, may have influenced participation. As with all self‑reported measures, the data may be affected by recall or social desirability bias; in a culturally sensitive context such as Bangladesh, this bias may be particularly pronounced, potentially leading to underreporting of tobacco use, especially among females. The recall periods for SHS exposure and tobacco advertising varied across items (e.g., past 7 days vs. past 30 days), consistent with GYTS measurement conventions [33], and were retained to maintain comparability with existing literature. However, the dichotomisation of regulatory knowledge variables and the use of varying recall periods across exposure measures may have reduced sensitivity to nuanced understanding and introduced potential recall bias. We acknowledge that dichotomising ordinal and Likert-scale variables results in loss of information and may reduce statistical power. This approach was chosen to ensure model stability, avoid sparse cell counts, and maintain comparability with prior GYTS-based analyses. Although the multivariable analyses adjusted for a broad range of sociodemographic and environmental factors, residual confounding cannot be excluded, particularly for unmeasured family-level and psychosocial influences. In addition, the study was not specifically powered to formally test interaction effects between sociodemographic characteristics and environmental exposures. As a result, potential effect modification may have been underestimated, and future studies with larger, probability-based samples should explore these interaction pathways in greater depth. It is important to emphasise that, given the cross-sectional nature of the study, the observed associations should be interpreted as correlational rather than causal, and reverse or bidirectional relationships cannot be ruled out. Additionally, the study’s urban focus may limit generalisability to rural areas, where tobacco use patterns could differ due to socio-economic and cultural factors, including the higher prevalence of smokeless tobacco use in rural settings. Furthermore, the sample may not be fully representative of all adolescents in Dhaka, particularly those not enrolled in school or attending private or English-medium institutions; the findings should, therefore, be interpreted with caution. Future research should explore these variations to provide a more comprehensive understanding of tobacco use across diverse populations.

## Conclusion

In conclusion, this study highlights the continued burden of tobacco use among adolescents in urban Bangladesh and underscores the importance of early, contextually grounded prevention strategies. The findings point to distinct gendered pathways into tobacco use, with peer and stress-related influences more prominent among boys and household-level influences particularly salient for girls, especially in relation to smokeless tobacco. Taken together, these findings have several concrete policy and practice implications. First, the strong influence of household tobacco use, particularly for smokeless tobacco initiation among girls, underscores the need for family-centred prevention strategies that extend beyond schools and explicitly target parental and caregiver tobacco use. Community-based interventions promoting smoke-free homes, coupled with cessation support for adult family members, may be particularly effective in reducing early exposure. Second, the persistent association between point-of-sale advertising and adolescent tobacco use highlights the need for stricter enforcement of existing advertising restrictions, including routine monitoring of retail outlets and penalties for non-compliance. Third, school-based tobacco prevention programmes should move beyond information provision alone and incorporate gender-sensitive components addressing peer pressure, stress coping, and social norms, particularly for boys. Integrating these elements into the national school curriculum could strengthen existing tobacco control efforts in Bangladesh.

## Supplementary Information

Supplementary Material 1.

Supplementary Material 2.

## Data Availability

Data can be provided upon a reasonable request from the corresponding author.
