# Supplementary material for: Prevalence and correlates of tobacco use among adolescents in Dhaka, Bangladesh: a cross-sectional study
Source: BMC Public Health. 2026 Feb 28;26:1123. doi: 10.1186/s12889-026-26821-7 (PMC13059170; doi:10.1186/s12889-026-26821-7)
Supplement: Supplementary file 1 — Supplementary Material 1. [file 12889_2026_26821_MOESM1_ESM.docx]

**LONDON’S GLOBAL UNIVERSITY**

**Supplementary file 1**

**Questionnaire**

**Instructions**

- Please read each question carefully before answering it.
- Choose the answer that best describes what you believe and feel to be correct.
- Some of the questions will ask about smoking **cigarettes**.
- Other questions may ask about **smoking tobacco** in general that includes cigarettes and other types of smoked tobacco products (e.g., Pipes, Cigars, Waterpipes/hookah/shisha, Bidis).
- Other questions may ask about using **smokeless tobacco** (e.g., Snuff, Chewing tobacco–zarda with pan, tobacco leaf, gul, panmasala–Betel quid with tobacco, Gutka), which is tobacco that is not smoked, but is sniffed through the nose, held in the mouth, or chewed.
- Other questions may ask about any tobacco use or any tobacco products – this includes smoking cigarettes, smoking tobacco other than cigarettes, and using smokeless tobacco.
- Finally, other questions may ask about your mental health and well-being.

**Section A: Demographic information**

The following questions ask for some background information about yourself.

| **SL** | **Question** | **Response options** |
| --- | --- | --- |
| A1 | Please provide the initials of your name |  |
| A2 | What is the name of your school/college? |  |
| A3 | In what class are you? | 1 = 8 (Eight)  2 = 9 (Nine)  3 = 10 (Ten)  4 = 11 (Eleven) |
| A4 | Please provide your Date of Birth (DD/MM/YYYY) | ……………………… |
| A5 | What is your sex? | 1 = Female  2 = Male |
| A6 | What is your religion? | 1 = Islam  2 = Hinduism  3 = Buddhism  4 = Christianity  5 = No religious belief |
| A7 | How many siblings do you have? | …………………… |
| A8 | What is the highest level of education of your mother? | 1 = No formal education  2 = Primary (1-5)  3 = Secondary (6-10)  4 = Higher Secondary (11-12)  5 = Undergraduate  6 = Graduate  7 = Doctoral |
| A9 | What is the highest level of education of your Father? | 1 = No formal education  2 = Primary (1-5)  3 = Secondary (6-10)  4 = Higher Secondary (11-12)  5 = Undergraduate  6 = Graduate  7 = Doctoral |
| A10 | During an average week, how much money do you have that you can spend on yourself, however you want? (in Taka) | ……………………..Taka |
| A11 | What is the current status of your housing tenure/ living accommodation? | 1 = School/College hostel  2 = Private hostel  3 = Rented house with friends  4 = Rented house with parents  5 = Owned property with parents  6 = Other |
| A11.6 | Show the field ONLY if: [a11] = '6'  Please specify… | ………………………….……..[Text] |

**Section B: Information about tobacco use**

The following questions ask about your use of tobacco.

| **SL** | **Question** | **Response options** |
| --- | --- | --- |
| B1 | Have you ever tried or experimented with cigarette smoking? | 1 = Yes  2 = No |
| B2 | How old were you when you first tried a cigarette? | ……….. |
| B3 | During the past 30 days, on how many days did you smoke cigarettes? | 1 = 0 days  2 = 1 or 2 days  3 = 3 to 5 days  4 = 6 to 9 days  5 = 10 to 19 days  6 = 20 to 29 days  7 = All 30 days |
| B4 | Please think about the days you smoked cigarettes during the past 30 days. How many times did you usually smoke per day? | 1 = Less than once per day  2 = Once per day  3 = 2 to 5 times per day  4 = 6 to 10 times per day  5 = 11 to 20 times per day  6 = More than 20 times per day |
| B5 | Have you ever tried or experimented with any form of smoked tobacco products other than cigarettes (pipes, cigars, waterpipes, hookah, shisha, bidis)? | 1 = Yes  2 = No |
| B6 | How old were you when you first tried that tobacco product? | …….. |
| B7 | During the past 30 days, on how many days did you use any form of smoked tobacco products other than cigarettes (e.g., pipes, cigars, waterpipes, hookah, shisha, bidis)? | 1 = 0 days  2 = 1 or 2 days  3 = 3 to 5 days  4 = 6 to 9 days  5 = 10 to 19 days  6 = 20 to 29 days  7 = All 30 days |
| B8 | Please think about the days you smoked tobacco products other than cigarettes (e.g., pipes, cigars, waterpipes, hookah, shisha, bidis) during the past 30 days. How many times did you usually smoke per day? | 1 = Less than once per day  2 = Once per day  3 = 2 to 5 times per day  4 = 6 to 10 times per day  5 = 11 to 20 times per day  6 = More than 20 times per day |
| B9 | Have you ever tried or experimented with any form of smokeless tobacco products (zorda with pan, tobacco leaf, gul, khaini, panmasala)? | 1 = Yes  2 = No |
| B10 | How old were you when you first tried using smokeless tobacco? | ………….. |
| B11 | During the past 30 days, on how many days did you use smokeless tobacco? | 1 = 0 days  2 = 1 or 2 days  3 = 3 to 5 days  4 = 6 to 9 days  5 = 10 to 19 days  6 = 20 to 29 days  7 = All 30 days |
| B12 | Please think about the days you used smokeless tobacco during the past 30 days. How many times did you usually use smokeless tobacco per day? | 1 = Less than once per day  2 = Once per day  3 = 2 to 5 times per day  4 = 6 to 10 times per day  5 = 11 to 20 times per day  6 = More than 20 times per day |
| B13 | What were the reasons behind your tobacco initiation?  (**Tick all that apply**) | 1 = Curiosity  2 = Tobacco makes me look more attractive  3 = Peer influence  4 = Tobacco users in the family  5 = Family conflict  6 = School factors  7 = Poor academic achievement  8 = Lack of self-esteem  9 = Anxiety/Desperation/Stress  10 = Availability of tobacco  11 = Exposure to advertisements/promotions at points of sale  12 = Seen people using tobacco when watched TV, videos or movies  13 = Others |
| B13.13 | Show the field ONLY if: [b13(13)] = '1'  Please specify ……. | ……………………..[Text] |
| B14 | Would you please let us know about your motivation toward stopping tobacco use?  (Please select the best option for you from the list) | 1 = I don't want to stop tobacco use  2 = I think I should stop tobacco use but don't really want to  3 = I want to stop tobacco use but haven't thought about when  4 = I REALLY want to stop tobacco use but I don't know when I will  5 = I want to stop tobacco use and hope soon  6 = I REALLY want to stop tobacco use and intend to in the next 3 months  7 = I REALLY want to stop tobacco use and intend to in the next month |
| B15 | During the past 12 months, have you ever try to stop tobacco use? | 1 = Yes  2 = No |
| B16 | Have you ever received help or advice to help you stop tobacco use?  (**Tick all that apply**) | 1 = No  2 = Yes, from a health care professional (e.g., doctor)  3 = Yes, from a teacher  4 = Yes, from a family member  5 = Yes, from a friend  6 = Yes, from a mobile app  7 = Yes, from an online source  8 = Yes, other (please specify: ______) |
| B16.8 | Show the field ONLY if: [b16] = '8'  Please specify: | ………………….…….[Text] |

**Section C: Information about second-hand tobacco smoke and exposure to the advertisements or promotions for tobacco**

The following questions ask about your exposure to other people’s smoking and tobacco advertisements.

| **SL** | **Question** | **Response options** |
| --- | --- | --- |
| C1 | During the past 7 days, on how many days has anyone smoked inside your home, in your presence? | 1 = 0 days  2 = 1 or 2 days  3 = 3 to 4 days  4 = 5 to 6 days  5 = 7 days |
| C2 | During the past 7 days, on how many days has anyone smoked in your presence, inside any enclosed public place, other than your home (such as: school, shops, restaurants, shopping malls, movie theatres, any office, inside bus, inside train)? | 1 = 0 days  2 = 1 or 2 days  3 = 3 to 4 days  4 = 5 to 6 days  5 = 7 days |
| C3 | During the past 7 days, on how many days has anyone smoked in your presence, at any outdoor public place (such as: playgrounds, sidewalks, entrance to buildings, parks, beaches, bus terminal, railway station)? | 1 = 0 days  2 = 1 or 2 days  3 = 3 to 4 days  4 = 5 to 6 days  5 = 7 days |
| C4 | During the past 30 days, did you see anyone smoke inside the school building or outside on school property? | 1 = Yes  2 = No |
|  | Advertisement |  |
| C5 | During the past 30 days, did you see any people using tobacco on TV, in videos, or movies? | 1 = Yes  2 = No  3 = I did not watch TV, videos, or movies in the past 30 days. |
| C6 | During the past 30 days, did you see any advertisements or promotions for tobacco products in retailers (such as: stores, shops, street vendors)? | 1 = Yes  2 = No  3 = I did not visit any retailers in the past 30 days. |
| C7 | During the past 30 days, did you see any advertisements or promotions for tobacco products on online social media (such as: Facebook, Instagram, YouTube, Twitter, etc.)? | 1 = Yes  2 = No  3 = I did not use any social-media in the past 30 days. |
|  | Promotion |  |
| C8 | Would you ever use or wear something that has a tobacco company or tobacco product name or picture on it such as a lighter, T-shirt, hat, or sunglasses? | 1 = Yes  2 = No  3 = Maybe |
| C9 | Do you have something (for example, T-shirt, pen, backpack) with a tobacco product band logo on it? | 1 = Yes  2 = No |
| C10 | Has a person working for a tobacco company ever offered you a free tobacco product? | 1 = Yes  2 = No |

**Section D: Knowledge, attitudes and beliefs about using tobacco products**

The following questions ask about your knowledge, attitudes and beliefs about using tobacco.

| **SL** | | **Question** | **Response options** | | |
| --- | --- | --- | --- | --- | --- |
| D1 | | If one of your friends offered you a tobacco product, would you use it? | 1 = Definitely not  2 = Probably not  3 = Probably yes  4 = Definitely yes | | |
| D2 | | At any time during the next 12 months do you think you will use any form of tobacco? | 1 = Definitely not  2 = Probably not  3 = Probably yes  4 = Definitely yes | | |
| D3 | Do you think the smoke from other people’s tobacco smoking is harmful to you? | | 1 = Definitely not  2 = Probably not  3 = Probably yes  4 = Definitely yes | | |
| D4 | | Once someone has started using tobacco, do you think it would be difficult for them to quit? | 1 = Definitely not  2 = Probably not  3 = Probably yes  4 = Definitely yes | | |
| D5 | | Do you think using tobacco helps people feel more comfortable or less comfortable at celebrations, parties, or in other social gatherings? | 1 = More comfortable  2 = Less comfortable  3 = No difference whether using tobacco or not | | |
| D6 | | Do you agree or disagree with the following: “I think I might enjoy using a tobacco product.” [Shown to anyone who answered Q B1=0 AND Q B5=0 AND Q B8-0] | 1 = Strongly agree  2 = Agree  3 = Disagree  4 = Strongly disagree | | |
| D7 | | Has anyone ever raised the topic of tobacco use with you? | 1 = Yes; go to next question  2 = No; go to “D10” | | |
| D8 | | Who raised the topic of tobacco use with you?  (**Tick all that apply**) | 1 = Friends  2 = Family member  3 = School teacher  4 = Health Professional  5 = Others (please specify……) | | |
| D8_5 | | Show the fi eld ONLY if: [d8(5)] = '1'  Please specify… | ………………. | | |
| D9 | | Did anyone tell you about the dangers/risks of tobacco use / provide anti-tobacco education? | 1 = Yes  2 = No | | |
| D10 | | Show the fi eld ONLY if: [d9] = '1'  Who told you about the dangers of tobacco use / provide anti-tobacco education?  (**Tick all that apply**) | 1 = Friends  2 = Family member  3 = School teacher  4 = Health Professional  5 = Others (please specify……) | | |
| D10_5 | | Show the fi eld ONLY if: [d10(5)] = '1'  Please specify...... | ………. | | |
| D11 | | I have knowledge about the rules and regulations for smoking and tobacco products use (control) in Bangladesh. | 1 = Strongly agree  2 = Agree  3 = Disagree  4 = Strongly disagree | | |
|  | | **Do the following statements are true or not true with your knowledge about the rules and regulations for smoking and tobacco products use (control) in Bangladesh?** | 1 = True | 2 = Not true | 3 = Don’t know |
| D12.1 | | No public places (e.g., educational institution, hospital, cinema hall), public areas (children park), and public transports shall be marked or identified as a smoking zone |  |  |  |
| D12.2 | | If movies contain a scene with tobacco products, the scene can be displayed without a written warning about the harmful effects of consuming tobacco products |  |  |  |
| D12.3 | | All types of tobacco promotional advertisement are banned by the law |  |  |  |
| D12.4 | | Selling tobacco products to the minors (less than 18 years old) is prohibited by law |  |  |  |
| D12.5 | | Either pictorial or written warning notice must cover the surface on the packets, packages, cartons or cans of tobacco products |  |  |  |
| D12.6 | | Violation of any provision of the tobacco control law in Bangladesh is protected with just a financial punishment |  |  |  |

**Section E: Information about mental health**

The following questions ask about your mental health conditions.

| E1 | How do you describe your general state of health now?  Or, In general, would you say your health is: | 1 = Very good  2 = Good  3 = Moderate (in between good and poor)  4 = Poor  5 = Very poor | | | |
| --- | --- | --- | --- | --- | --- |
| **E2** | **Over the last 2 weeks, how often have you been bothered by any of the following problems?** | **Not at all**  **(0)** | **Several days**  **(1)** | **More than half the days**  **(2)** | **Nearly every day (3)** |
| E2.1 | Feeling nervous, anxious |  |  |  |  |
| E2.2 | Not being able to sleep or control worrying |  |  |  |  |
| E2.3 | Worrying too much about different things |  |  |  |  |
| E2.4 | Trouble relaxing |  |  |  |  |
| E2.5 | Being so restless that it is hart to sit still |  |  |  |  |
| E2.6 | Becoming easily annoyed or irritable |  |  |  |  |
| E2.7 | Feeling afraid as if something awful might happen |  |  |  |  |
| E3.1 | Little interest or pleasure in doing things |  |  |  |  |
| E3.2 | Feeling down, depressed, or hopeless? |  |  |  |  |
| E3.3 | Trouble falling or staying asleep, or sleeping too much |  |  |  |  |
| E3.4 | Feeling tired or having little energy |  |  |  |  |
| E3.5 | Poor appetite or overeating |  |  |  |  |
| E3.6 | Feeling bad about yourself - or that you are a failure or have let yourself or your family down |  |  |  |  |
| E3.7 | Trouble concentrating on things, such as reading the newspaper or watching television |  |  |  |  |
| E3.8 | Moving or speaking so slowly that other people could have noticed  Or, the opposite - being so fidgety or restless that you have been moving around a lot more than usual? |  |  |  |  |
| E3.9 | Thoughts that you would be better off dead, or of hurting yourself in some way? |  |  |  |  |

**Section F: Qualitative part**

The following questions will seek your opinion about what a possible tobacco use prevention programme should look like.

Consider a scenario where somebody is trying to develop **a training programme for tobacco use prevention** among adolescents like you and implement it in school settings. Please describe the answer to the following questions in your way.

F1. How many sessions would you prefer to receive on this, and how long should each session be?

F2. What information, tasks or activities, do you think, would most likely prevent you or other adolescents to start using tobacco?

F3. Which of the following components would you like to see in the training sessions and why? (Mention all that apply)

1. Graphical material (poster, information cards, and flyers); (_______why)
2. Motivational videos; (_______why)
3. Quiz activities; (_______why)
4. Didactic material (e.g., crossword puzzles); (_______why)
5. Group dividing activities; (_______why)
6. Games; (_______why)
7. Music and songs; (_______why)
8. Religious story; (_______why)
9. Others; (please specify with reasons: _________)

F4. Would you prefer to see a friend of yours, or a teacher of your school, or an outsider expert in the subject matter as the trainer of the training programme? (please mention the reasons for your preference)

F5. Do you think a tobacco use prevention programme is needed in your school?

1 = Yes

2 = No

F5_1 (Why/why not): …………………………………………….

F6. Do you think such a tobacco use prevention programme should also address household tobacco use simultaneously?

1 = Yes

2 = No

F6_1 (Why/why not): …………………………………………….

F7. What possible difficulties might you face in participating in the training programme?

**End the questionnaire.**

**Thank you very much.**

**Submit option**
