# Supplementary material for: Prevalence and correlates of tobacco use among adolescents in Dhaka, Bangladesh: a cross-sectional study
Source: BMC Public Health. 2026 Feb 28;26:1123. doi: 10.1186/s12889-026-26821-7 (PMC13059170; doi:10.1186/s12889-026-26821-7)
Supplement: Supplementary file 2 — Supplementary Material 2. [file 12889_2026_26821_MOESM2_ESM.docx]

**Supplementary file 2**

**Supplementary table S1: Self-reported reasons for tobacco initiation of school adolescents in Dhaka, stratified by sex (N=415)**

| **Characteristics** | | **Total, % (n)** | **Sex** | | |
| --- | --- | --- | --- | --- | --- |
|  | |  | **Female, % within sex (n)** | **Male, % within sex (n)** | **χ^2^-value (p-value)** |
| Curiosity | |  |  |  |  |
|  | Yes | 60.7 (252) | 60.4 (67) | 60.9 (185) | 0.008 (0.927) |
|  | No | 39.3 (163) | 39.6 (44) | 39.1 (119) |  |
| Peer influence | |  |  |  |  |
|  | Yes | 22.2 (92) | 14.4 (16) | 25.0 (76) | **5.281 (0.022)** |
|  | No | 77.8 (323) | 85.6 (95) | 75.0 (228) |  |
| Anxiety/Desperation/Stress | |  |  |  |  |
|  | Yes | 22.2 (92) | 10.8 (12) | 26.3 (80) | **11.329 (0.001)** |
|  | No | 77.8 (323) | 89.2 (99) | 73.7 (224) |  |
| Tobacco users in the family | |  |  |  |  |
|  | Yes | 16.9 (70) | 67.6 (75) | 11.2 (34) | **26.180 (<0.001)** |
|  | No | 83.1 (345) | 32.4 (36) | 88.8 (270) |  |
| Seen people using tobacco when watched TV, videos or movies | |  |  |  |  |
|  | Yes | 11.1 (46) | 9.9 (11) | 11.5 (35) | 0.212 (0.645) |
|  | No | 88.9 (369) | 90.1 (100) | 88.5 (269) |  |
| Poor academic achievement | |  |  |  |  |
|  | Yes | 10.6 (44) | 6.3 (7) | 12.2 (37) | 2.951 (0.086) |
|  | No | 89.4 (371) | 93.7 (104) | 87.8 (267) |  |
| Availability of tobacco | |  |  |  |  |
|  | Yes | 10.6 (44) | 8.1 (9) | 11.5 (35) | 0.995 (0.319) |
|  | No | 89.4 (371) | 91.9 (102) | 88.5 (269) |  |
| Family conflict | |  |  |  |  |
|  | Yes | 7.7 (32) | 3.6 (4) | 9.2 (28) | 3.592 (0.063)^a^ |
|  | No | 92.3 (383) | 96.4 (107) | 90.8 (276) |  |
| School factors | |  |  |  |  |
|  | Yes | 7.5 (31) | 4.5 (5) | 8.6 (26) | 1.928 (0.207)^a^ |
|  | No | 92.5 (384) | 95.5 (106) | 91.4 (278) |  |
| Tobacco makes me look more attractive | |  |  |  |  |
|  | Yes | 6.5 (27) | 3.6 (4) | 7.6 (23) | 2.099 (0.181)^a^ |
|  | No | 93.5 (388) | 96.4 (107) | 92.4 (281) |  |
| Lack of self-esteem | |  |  |  |  |
|  | Yes | 5.5 (23) | 2.7 (3) | 6.6 (20) | 2.334 (0.151)^a^ |
|  | No | 94.5 (392) | 97.3 (108) | 93.4 (284) |  |
| Exposure to advertisements/promotions at points of sale | |  |  |  |  |
|  | Yes | 2.7 (11) | 1.8 (2) | 3.0 (9) | 0.423 (0.735)^a^ |
|  | No | 97.3 (404) | 98.2 (109) | 97.0 (295) |  |
| Other | |  |  |  |  |
|  | Yes | 5.8 (24) | 8.1 (9) | 4.9 (15) | 1.503 (0.220) |
|  | No | 94.2 (391) | 91.9 (102) | 95.1 (289) |  |

^a^ p-value is based on Fisher’s exact test

**Supplementary table S2: Association of demographic characteristics and key correlates with smoked tobacco use behaviour among school adolescents in Dhaka, Bangladesh**

| **Variable** | | **Ever smoked tobacco use ^a^** | | | |  | **Current smoked tobacco use ^b^** | | | |
| --- | --- | --- | --- | --- | --- | --- | --- | --- | --- | --- |
|  |  | **Unadjusted model** | | **Adjusted model ^c^** | |  | **Unadjusted model** | | **Adjusted model ^d^** | |
|  | | **OR**  **(95% CI)** | **p-value** | **AOR**  **(95% CI)** | **p-value** |  | **OR (95% CI)** | **p-value** | **AOR (95% CI)** | **p-value** |
| **Demographics** | |  |  |  |  |  |  |  |  |  |
| Sex | |  |  |  |  |  |  |  |  |  |
|  | Male | 4.67  (3.38–6.45) | **<0.001** | 3.59  (2.46–5.24) | **<0.001** |  | 12.21 (6.59–22.64) | **<0.001** | 7.20 (3.56–14.55) | **<0.001** |
|  | Female | 1.00 |  | 1.00 |  |  | 1.00 |  | 1.00 |  |
| Age (in years) | |  |  |  |  |  |  |  |  |  |
|  | 17-18 | 5.43  (3.05–9.68) | **<0.001** | 2.59  (1.40–4.80) | **0.003** |  | 8.55 (3.46–21.13) | **<0.001** | 3.24 (1.25–8.44) | **0.016** |
|  | 14-16 | 1.85  (1.02–3.35) | **0.042** | 1.22  (0.65–2.28) | 0.540 |  | 2.19 (0.86–5.57) | **0.100** | 1.24 (0.46–3.33) | 0.669 |
|  | 11-13 | 1.00 |  | 1.00 |  |  | 1.00 |  | 1.00 |  |
| Living accommodation | |  |  |  |  |  |  |  |  |  |
|  | Hostel & other | 2.54 (1.73–3.74) | **<0.001** | 1.38 (0.88–2.17) | 0.166 |  | 3.87 (2.42–6.20) | **<0.001** | 2.32 (1.30–4.11) | **0.004** |
|  | Owned house with parents | 1.67 (1.28–2.18) | **<0.001** | 1.43 (1.04–1.95) | **0.026** |  | 2.23 (1.57–3.20) | **<0.001** | 2.18 (1.40–3.38) | **<0.001** |
|  | Rented house with parents | 1.00 |  | 1.00 |  |  | 1.00 |  | 1.00 |  |
| Monthly pocket money expenditure | |  |  |  |  |  |  |  |  |  |
|  | Middle and high | 2.73 (2.13–3.50) | **<0.001** | 1.70 (1.26–2.29) | **<0.001** |  | 3.33 (2.41–4.59) | **<0.001** | 1.77 (1.19–2.64) | **0.005** |
|  | Low and lower middle | 1.00 |  | 1.00 |  |  | 1.00 |  | 1.00 |  |
| Father’s educational level | |  |  |  |  |  |  |  |  |  |
|  | University education | 0.91 (0.70–1.17) | 0.450 |  |  |  | 0.93 (0.67–1.30) | 0.680 |  |  |
|  | No university | 1.00 |  |  |  |  | 1.00 |  |  |  |
| Mother’s educational level | |  |  |  |  |  |  |  |  |  |
|  | University education | 1.18 (0.93–1.51) | 0.181 |  |  |  | 1.36 (0.99–1.86) | 0.061 |  |  |
|  | No university | 1.00 |  |  |  |  | 1.00 |  |  |  |
| **Exposure to SHS, advertisement, or promotion** | |  |  |  |  |  |  |  |  |  |
| Exposed to SHS at home | |  |  |  |  |  |  |  |  |  |
|  | Yes | 2.35 (1.80–3.08) | **<0.001** | 1.74 (1.27–2.39) | **<0.001** |  | 2.52 (1.79–3.55) | **<0.001** | 1.62 (1.07–2.47) | **0.023** |
|  | No | 1.00 |  | 1.00 |  |  | 1.00 |  | 1.00 |  |
| Exposed to SHS in an enclosed public place | |  |  |  |  |  |  |  |  |  |
|  | Yes | 2.20 (1.49–3.27) | **<0.001** | 1.88 (1.13–3.13) | **0.015** |  | 2.45 (1.42–4.21) | **0.001** | 2.18 (1.03–4.62) | **0.043** |
|  | No | 1.00 |  | 1.00 |  |  | 1.00 |  | 1.00 |  |
| Exposed to SHS in an outdoor public place | |  |  |  |  |  |  |  |  |  |
|  | Yes | 2.08 (1.36–3.19) | **<0.001** | 1.17 (0.69–1.99) | 0.563 |  | 2.42 (1.33–4.41) | **0.004** | 1.20 (0.55–2.61) | 0.650 |
|  | No | 1.00 |  | 1.00 |  |  | 1.00 |  | 1.00 |  |
| Witnessed tobacco use in/outside of school premises | |  |  |  |  |  |  |  |  |  |
|  | Yes | 2.36 (1.81–3.08) | **<0.001** | 1.52 (1.12–2.08) | **0.008** |  | 3.11 (2.16–4.49) | **<0.001** | 1.87 (1.20–2.90) | **0.005** |
|  | No | 1.00 |  | 1.00 |  |  | 1.00 |  | 1.00 |  |
| Observed tobacco use on TV, in videos, or in movies | |  |  |  |  |  |  |  |  |  |
|  | Yes | 2.05 (1.48–2.85) | **<0.001** | 1.39 (0.94–2.04) | 0.096 |  | 2.37 (1.50–3.72) | **<0.001** | 1.56 (0.90–2.73) | 0.117 |
|  | No | 1.00 |  | 1.00 |  |  | 1.00 |  | 1.00 |  |
| Exposed to point-of-sale tobacco advertisement or promotion | |  |  |  |  |  |  |  |  |  |
|  | Yes | 2.14 (1.64–2.79) | **<0.001** | 1.39 (1.00–1.92) | **0.050** |  | 2.29 (1.63–3.21) | **<0.001** | 1.17 (0.76–1.79) | 0.485 |
|  | No | 1.00 |  | 1.00 |  |  | 1.00 |  | 1.00 |  |
| Exposed to online (social media platforms) tobacco advertisement or promotion | |  |  |  |  |  |  |  |  |  |
|  | Yes | 1.54 (1.13–2.09) | **0.006** | 0.74 (0.50–1.08) | 0.120 |  | 1.76 (1.21–2.58) | **0.004** | 0.69 (0.42–1.16) | 0.161 |
|  | No | 1.00 |  | 1.00 |  |  | 1.00 |  | 1.00 |  |
| Ever used non-tobacco products with tobacco company or tobacco product name, brand logo or picture on it | |  |  |  |  |  |  |  |  |  |
|  | Yes | 4.04 (2.72–6.00) | **<0.001** | 1.86 (1.04–3.34) | **0.038** |  | 5.16 (3.29–8.09) | **<0.001** | 2.05 (1.00–4.20) | 0.051 |
|  | No | 1.00 |  | 1.00 |  |  | 1.00 |  | 1.00 |  |
| Currently using non-tobacco products with tobacco company or tobacco product name, brand logo or picture on it | |  |  |  |  |  |  |  |  |  |
|  | Yes | 3.77 (2.38–5.98) | **<0.001** | 1.13 (0.57–2.25) | 0.730 |  | 5.34 (3.21–8.88) | **<0.001** | 1.56 (0.68–3.57) | 0.295 |
|  | No | 1.00 |  | 1.00 |  |  | 1.00 |  | 1.00 |  |
| Got offer and/or received complimentary tobacco products | |  |  |  |  |  |  |  |  |  |
|  | Yes | 3.79 (2.56–5.60) | **<0.001** | 1.44 (0.89–2.31) | 0.138 |  | 6.25 (4.06–9.60) | **<0.001** | 2.25 (1.30–3.87) | **0.004** |
|  | No | 1.00 |  | 1.00 |  |  | 1.00 |  | 1.00 |  |
| **Tobacco education and regulatory knowledge** | |  |  |  |  |  |  |  |  |  |
| Have received health education on the risks of tobacco use | |  |  |  |  |  |  |  |  |  |
|  | Yes | 0.63 (0.45–0.88) | **0.007** | 0.62 (0.42–0.91) | **0.014** |  | 0.67 (0.43–1.05) | 0.079 |  |  |
|  | No | 1.00 |  | 1.00 |  |  | 1.00 |  |  |  |
| Have knowledge about country’s tobacco control rules and regulations | |  |  |  |  |  |  |  |  |  |
|  | Yes | 0.50 (0.37–0.68) | **<0.001** | 0.65 (0.46–0.91) | **0.011** |  | 0.47 (0.32–0.69) | **<0.001** | 0.61 (0.40–0.94) | **0.026** |
|  | No | 1.00 |  | 1.00 |  |  | 1.00 |  | 1.00 |  |

Note. OR – odds ratio; AOR – adjusted odds ratio; CI – confidence interval; SHS – second hand smoke.

^a^ Estimates are based on binary logistic regression with ever smoked tobacco use as dependent variable.

^b^ Estimates are based on binary logistic regression with current smoked tobacco use as dependent variable.

^c^ Adjusted for all significant variables in the unadjusted model of ever smoked tobacco use.

^d^ Adjusted for all significant variables in the unadjusted model of current smoked tobacco use.

**Supplementary table S3: Association of demographic characteristics and key correlates with smokeless tobacco use behaviour among school adolescents in Dhaka, Bangladesh**

| **Variable** | | **Ever smokeless tobacco use ^a^** | | | |  | **Current smokeless tobacco use ^b^** | | | |
| --- | --- | --- | --- | --- | --- | --- | --- | --- | --- | --- |
|  |  | **Unadjusted model** | | **Adjusted model ^c^** | |  | **Unadjusted model** | | **Adjusted model ^d^** | |
|  | | **OR (95% CI)** | **p-value** | **AOR (95% CI)** | **p-value** |  | **OR (95% CI)** | **p-value** | **AOR (95% CI)** | **p-value** |
| **Demographics** | |  |  |  |  |  |  |  |  |  |
| Sex | |  |  |  |  |  |  |  |  |  |
|  | Male | 1.22 (0.91–1.64) | 0.186 |  |  |  | 2.18 (1.15–4.14) | **0.017** | 1.25 (0.58–2.66) | 0.569 |
|  | Female | 1.00 |  |  |  |  | 1.00 |  | 1.00 |  |
| Age (in years) | |  |  |  |  |  |  |  |  |  |
|  | 17-18 | 2.87 (1.51–5.43) | **0.001** | 2.37 (1.14–4.93) | **0.021** |  | 4.77 (1.13–20.14) | **0.033** | 1.97 (0.43–9.10) | 0.387 |
|  | 14-16 | 2.33 (1.57–3.09) | **0.009** | 2.60 (1.28–5.30) | **0.008** |  | 2.27 (0.52–9.82) | 0.274 | 1.53 (0.33–7.01) | 0.588 |
|  | 11-13 | 1.00 |  | 1.00 |  |  | 1.00 |  | 1.00 |  |
| Living accommodation | |  |  |  |  |  |  |  |  |  |
|  | Hostel & other | 2.18 (1.41–3.38) | **<0.001** | 1.93 (1.19–3.13) | **0.008** |  | 4.33 (1.92–9.77) | **<0.001** | 4.12 (1.57–10.79) | **0.004** |
|  | Owned house with parents | 1.19 (0.87–1.64) | 0.275 | 1.15 (0.82–1.63) | 0.423 |  | 2.27 (1.18–4.37) | **0.014** | 2.41 (1.14–5.11) | **0.022** |
|  | Rented house with parents | 1.00 |  | 1.00 |  |  | 1.00 |  | 1.00 |  |
| Monthly pocket money expenditure | |  |  |  |  |  |  |  |  |  |
|  | Middle and high | 1.68 (1.24–2.26) | **<0.001** | 1.31 (0.93–1.84) | 0.127 |  | 2.99 (1.68–5.31) | **<0.001** | 1.65 (0.84–3.24) | 0.150 |
|  | Low and lower middle | 1.00 |  | 1.00 |  |  | 1.00 |  | 1.00 |  |
| Father’s educational level | |  |  |  |  |  |  |  |  |  |
|  | University education | 0.97 (0.72–1.31) | 0.843 |  |  |  | 0.88 (0.49–1.60) | 0.682 |  |  |
|  | No university | 1.00 |  |  |  |  | 1.00 |  |  |  |
| Mother’s educational level | |  |  |  |  |  |  |  |  |  |
|  | University education | 1.18 (0.88–1.57) | 0.275 |  |  |  | 1.38 (0.78–2.45) | 0.271 |  |  |
|  | No university | 1.00 |  |  |  |  | 1.00 |  |  |  |
| **Exposure to SHS, advertisement, or promotion** | |  |  |  |  |  |  |  |  |  |
| Exposed to SHS at home | |  |  |  |  |  |  |  |  |  |
|  | Yes | 1.93 (1.40–2.66) | **<0.001** | 1.40 (0.99–1.99) | 0.058 |  | 4.27 (2.33–7.83) | **<0.001** | 2.37 (1.21–4.65) | **0.012** |
|  | No | 1.00 |  | 1.00 |  |  | 1.00 |  | 1.00 |  |
| Exposed to SHS in an enclosed public place | |  |  |  |  |  |  |  |  |  |
|  | Yes | 1.68 (1.10–2.58) | **0.017** | 1.28 (0.80–2.04) | 0.305 |  | 2.02 (0.79–5.15) | 0.142 |  |  |
|  | No | 1.00 |  | 1.00 |  |  | 1.00 |  |  |  |
| Exposed to SHS in an outdoor public place | |  |  |  |  |  |  |  |  |  |
|  | Yes | 1.49 (0.95–2.34) | 0.081 |  |  |  | 8.72 (1.20–63.54) | **0.033** | 6.29 (0.83–47.78) | 0.075 |
|  | No | 1.00 |  |  |  |  | 1.00 |  | 1.00 |  |
| Witnessed tobacco use in/outside of school premises | |  |  |  |  |  |  |  |  |  |
|  | Yes | 1.79 (1.32–2.42) | **<0.001** | 1.13 (0.80–1.58) | 0.489 |  | 1.95 (1.05–3.65) | **0.035** | 0.77 (0.38–1.57) | 0.472 |
|  | No | 1.00 |  | 1.00 |  |  | 1.00 |  | 1.00 |  |
| Observed tobacco use on TV, in videos, or in movies | |  |  |  |  |  |  |  |  |  |
|  | Yes | 1.74 (1.20–2.52) | **0.003** | 1.20 (0.80–1.82) | 0.374 |  | 2.59 (1.09–6.16) | **0.032** | 1.34 (0.52–3.48) | 0.549 |
|  | No | 1.00 |  | 1.00 |  |  | 1.00 |  | 1.00 |  |
| Exposed to point-of-sale tobacco advertisement or promotion | |  |  |  |  |  |  |  |  |  |
|  | Yes | 2.92 (2.16–3.96) | **<0.001** | 2.27 (1.61–3.22) | **<0.001** |  | 4.46 (2.42–8.23) | **<0.001** | 2.85 (1.40–5.82) | **0.004** |
|  | No | 1.00 |  | 1.00 |  |  | 1.00 |  | 1.00 |  |
| Exposed to online (social media platforms) tobacco advertisement or promotion | |  |  |  |  |  |  |  |  |  |
|  | Yes | 1.73 (1.23–2.45) | **0.002** | 0.95 (0.64–1.42) | 0.811 |  | 3.21 (1.73–5.97) | **<0.001** | 1.24 (0.58–2.62) | 0.582 |
|  | No | 1.00 |  | 1.00 |  |  | 1.00 |  | 1.00 |  |
| Ever used non-tobacco products with tobacco company or tobacco product name, brand logo or picture on it | |  |  |  |  |  |  |  |  |  |
|  | Yes | 3.29 (2.08–5.19) | **<0.001** | 1.32 (0.69–2.55) | 0.405 |  | 6.50 (3.20–13.21) | **<0.001** | 1.47 (0.47–4.54) | 0.506 |
|  | No | 1.00 |  | 1.00 |  |  | 1.00 |  | 1.00 |  |
| Currently using non-tobacco products with tobacco company or tobacco product name, brand logo or picture on it | |  |  |  |  |  |  |  |  |  |
|  | Yes | 3.78 (2.27–6.29) | **<0.001** | 1.63 (0.79–3.39) | 0.189 |  | 8.36 (3.99–17.51) | **<0.001** | 3.15 (0.96–10.38) | 0.059 |
|  | No | 1.00 |  | 1.00 |  |  | 1.00 |  | 1.00 |  |
| Got offer and/or received complimentary tobacco products | |  |  |  |  |  |  |  |  |  |
|  | Yes | 2.62 (1.63–4.22) | **<0.001** | 1.34 (0.77–2.33) | 0.294 |  | 4.01 (1.82–8.82) | **<0.001** | 0.95 (0.35–2.58) | 0.917 |
|  | No | 1.00 |  | 1.00 |  |  | 1.00 |  | 1.00 |  |
| **Tobacco education and regulatory knowledge** | |  |  |  |  |  |  |  |  |  |
| Have received health education on the risks of tobacco use | |  |  |  |  |  |  |  |  |  |
|  | Yes | 0.59 (0.41–0.86) | **0.006** | 0.64 (0.43–0.96) | **0.030** |  | 0.29 (0.15–0.55) | **<0.001** | 0.27 (0.13–0.56) | **<0.001** |
|  | No | 1.00 |  | 1.00 |  |  | 1.00 |  | 1.00 |  |
| Have knowledge about country’s tobacco control rules and regulations | |  |  |  |  |  |  |  |  |  |
|  | Yes | 0.42 (0.30–0.59) | **<0.001** | 0.51 (0.36–0.73) | **<0.001** |  | 0.36 (0.19–0.68) | **0.002** | 0.56 (0.28–1.13) | 0.106 |
|  | No | 1.00 |  | 1.00 |  |  | 1.00 |  | 1.00 |  |

Note. OR – odds ratio; AOR – adjusted odds ratio; CI – confidence interval; SHS – second hand smoke.

^a^ Estimates are based on binary logistic regression with ever smokeless tobacco use as dependent variable.

^b^ Estimates are based on binary logistic regression with current smokeless tobacco use as dependent variable.

^c^ Adjusted for all significant variables in the unadjusted model of ever smokeless tobacco use.

^d^ Adjusted for all significant variables in the unadjusted model of current smokeless tobacco use.

Not estimable: Estimate could not be calculated due to insufficient observations or data imbalance.
